# Accessibility of Medicines for Children: A Systematic Review

**DOI:** 10.3389/fphar.2021.691606

**Published:** 2021-08-05

**Authors:** Zhe Chen, Siyu Li, Linan Zeng, Yan Liu, Miao Zhang, Imti Choonara, Lingli Zhang

**Affiliations:** ^1^ Department of Pharmacy, West China Second University Hospital, Sichuan University, Chengdu, China; ^2^ Evidence-based Pharmacy Center, West China Second University Hospital, Sichuan University, Chengdu, China; ^3^ Key Laboratory of Birth Defects and Related Diseases of Women and Children, Ministry of Education, Chengdu, China; ^4^ West China School of Pharmacy, Sichuan University, Chengdu, China; ^5^ West China School of Medicine, Sichuan University, Chengdu, China; ^6^ Academic Division of Child Health, Derbyshire Children’s Hospital, University of Nottingham, Derby, United Kingdom

**Keywords:** children, medicine, accessibility, systematic review, availability, price, affordability

## Abstract

**Background:** Accessibility of medicines for children is a matter of global concern. Medicines prescribed for children are often off-label. To formulate appropriate policies and undertake necessary interventions to improve access to medicines for children, it is necessary to evaluate the accessibility of medicines for children. However, there is no systematic review of the medicine accessibility for children.

**Methods:** Relevant studies were identified through searching Pubmed, Embase, CNKI, Wanfang, VIP, World Health Organization website, and Health Action International website. Besides, the references of included studies as a supplementary search were read. We extracted the basic information of articles (the first author, published year, the name of journal, research institution, etc.), the basic study characteristics (survey area, survey time, survey method, survey medicine lists, the number of medicine outlets surveyed, etc.), and the study results (the current situation of the accessibility of medicines for children, including the availability, price, and affordability of medicines for children, etc.). Two reviewers independently selected studies and extracted the data. Descriptive analysis methods to analyze the current situation of the accessibility of children’s medicines were used.

**Results:** A total of 18 multicenter cross-sectional studies were included in this systematic review, which were from low-income and middle-income countries. Seventeen studies (17/18, 94.4%) used the WHO/Health Action International (HAI) medicine price methodology to investigate the accessibility of medicines for children. Fifteen studies (15/18, 83.3%) were selected to investigate medicines based on the WHO Model List of Essential Medicines for Children (WHO EMLc). In the public sectors, the availability of originator brands (OBs) ranged from 0 to 52.0%, with a median of 24.2%, and the availability of lowest-priced generics (LPGs) ranged from 17.0 to 72.6%, with a median of 38.1%. In the private sectors, the availability of OBs ranged from 8.9 to 80%, with a median of 21.2%. The availability of LPGs ranged from 20.6 to 72.2%, with a median of 35.9%. In most regions, the availability of OBs in the private sectors was higher than in the public sectors. Collectively, in the price of medicines for children, the median price ratio (MPR) of the OBs in the public sectors and private sectors were much higher than that of the LPGs. And the affordability of the LPGs in the public sectors and private sectors was higher than that of originator brands (OBs).

**Conclusion:** The availability of medicines for children is low in both the public sectors and private sectors in low-income and middle-income countries. The MPR of originator brands (OBs) is higher than that of lowest-priced generics (LPGs), and the most lowest-priced generics (LPGs) have better affordability.

## Introduction

In 2019, an estimated 5.2 million children under 5 years old died mostly from preventable and treatable causes. The leading causes of death in children under 5 years of age are preterm birth complications, birth asphyxia/trauma, pneumonia, congenital anomalies, diarrhea, and malaria. However, more than half of deaths associated with the potential complications can be prevented or treated with access to medicine treatment (World Health Organization, 2020). Unfortunately, the availability of medicines for children is low.

Nonavailability of child-size medicines encourages the use of adult dosage forms, splitting them into parts before giving them to a child. This practice is not scientific and is far from rational since children are not just miniature adults. Their anatomical and physiological characteristics are very different from those of adults. To extremely avoiding the risks of medicines use in children, medicines used by children should suit a children’s size, age, physiologic condition, and treatment requirements (Zhang et al., 2013; Ivanovska et al., 2014; Batchelor and Marriott, 2015; Zhang et al., 2017). To satisfy children’s basic medicine needs, WHO has published seven editions of the WHO Model List of Essential Medicines for Children (EMLc) (World Health Organization, 2019a) from October 2007 to July 2019. Besides, in 2009, the WHO launched the “Better Medicines for Children” initiative and “Make Medicines Child Size” campaign to enhance the accessibility of safe, effective, and quality medicines for children by promoting awareness and action through research, regulatory measures, and policy changes (Finney, 2019).

So far, a large number of researchers around the world have conducted a series of studies to evaluate the accessibility of medicines, and most of the studies have adapted the WHO/HAI standardized methodology. According to the data recorded by the WHO website, as of 2019, 76 studies from 55 countries world-wide have been conveyed by using the WHO/HAI standardized methodology (World Health Organization, 2019b). Among them, seven studies have evaluated the accessibility of medicines on essential medicines for children, and the results demonstrated that the overall availability of medicines for children was low (World Health Organization, 2019a).

To improve access to medicines for children, it is necessary to evaluate the accessibility of medicines for children. However, there is no systematic review of the medicine accessibility for children. Therefore, this review aims to systematically evaluate the current situation of children’s medicines accessibility globally and provide evidence for relevant organizations to formulate appropriate policies and undertake necessary interventions to improve medicine accessibility for children.

## Methods

### Search Strategy

Data sourced from Pubmed, Embase, CNKI, Wanfang, VIP, World Health Organization website, and Health Action International website were collected. References of the included studies were also searched for a supplementary study. The search strategy was adjusted specifically for each database and included a combination of medical subject headings and free text terms for (“child*” or “pediatri*”) and (“medicine*” or “drug*” or “medication*”) and (“access*” or “availab*” or “afford*” or “price*”). The deadline for all retrieval was January 2021.

### Inclusion Criteria

The following studies were included: 1) Participants: children (0–18 years), which were classified according to the International Conference on Harmonisation of Technical Requirements for Registration of Pharmaceuticals for Human Use Criteria (ICH, 2000). 2) Intervention: medicines for children. 3) Outcomes: the current situation of children’s medicines accessibility, including availability, price, or affordability. 4) Type of study: no limit.

### Exclusion Criteria

The following studies were excluded: 1) non-Chinese and non-English literature, 2) editorials, reviews, conference abstracts, and any unobtainable full-texts, 3) only investigated the availability of a single medicine or medicine dosage form.

### Risk of Bias Assessment

Combie scale (CROMBIE I, 1996) to evaluate the risk of bias in included studies were used from the following seven aspects: 1) the design was scientific; 2) the data collection strategy was reasonable; 3) the sample response rate was reported; 4) the sample was representative of the population; 5) the study purpose and method were reasonable; 6) the test power was reported; 7) the statistical method was reasonable. Combie scale categorized each item with “yes,” “no” and “unclear,” and gave them “1,” “0” and “0.5” points. If an item was not suitable for a study, it would be expressed as “-” and one point was recorded. The total score of this scale was 7.0 points, 6.0–7.0 was A-level (the quality of a study is high), 4.0–5.5 was B-level (the quality of a study is medium), and less than 4.0 was C-level (the quality of a study is low). Two researchers assessed the risk of bias independently, and their disputes reached an agreement by discussing, or deciding by another researcher.

### Data Extraction

Two independent reviewers screened all the titles and abstracts to determine potential eligible studies. They independently applied the eligibility criteria to perform the final selection. When discrepancies occurred between both reviewers, they would discuss and identify the reasons to either include or exclude the studies and then make the final decision. If they could not reach an agreement, the final decision would be based on a third reviewer. Two reviewers independently extracted data from included studies and cross-checked them. The extracted data included: 1) the basic information of articles (the first author, published year, the name of the published journal, research institution, etc.); 2) the basic study characteristics (survey area, survey time, research method, survey medicine lists, the number of medicine outlets surveyed, etc.); 3) the study results (the current situation of the accessibility of medicines for children, including the availability, price, and affordability of medicines for children, etc.).

### Data Analysis

We used descriptive analysis methods to analyze the current situation of accessibility of children’s medicines. For studies that directly reported the availability, price, and affordability of children’s medicines in general, we extracted relevant data. For other studies that only reported the original data of a single medicine, we made corresponding calculations based on the original data provided. Since the percentage of the medicine availability and the price of medicines do not follow a normal distribution, we used the median and quartile range to represent them.

## Results

### Characteristics of the Included Studies

**FIGURE 1:**
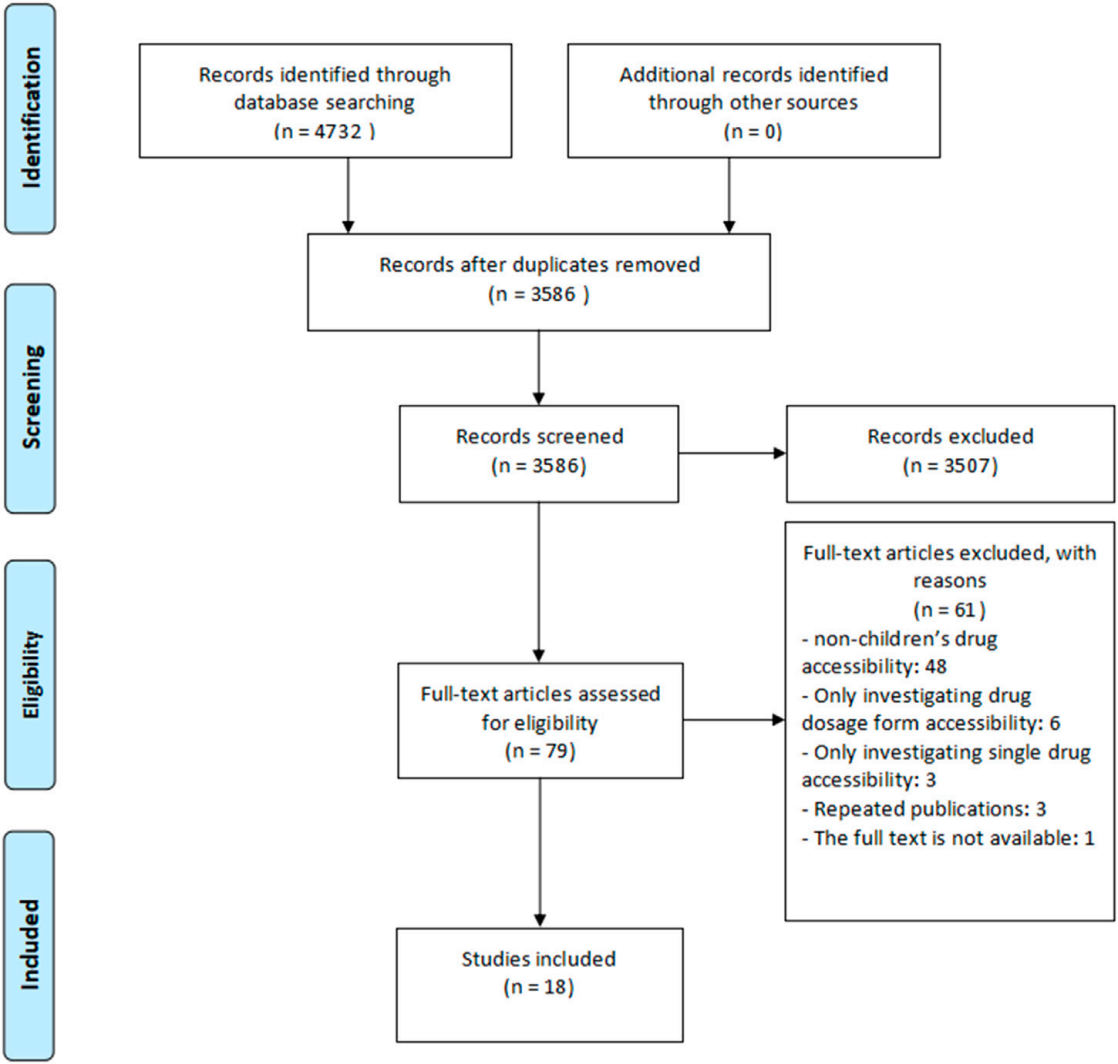
Flow diagram of the study selection process.

For initial screening, 4,732 records were identified. After removing duplicates and irrelevant records by screening for titles and abstracts, 79 studies were assessed for eligibility at full-text screening. Eventually, a total of 18 studies were enrolled in this systematic review (Figure 1).

The 18 included studies were all multicenter cross-sectional surveys and were conducted from 2009 to 2019 (Balasubramaniam et al., 2011; Anson et al., 2012; Ravindran et al., 2012; Balasubramaniam et al., 2014; Nsabagasani et al., 2015; Swain et al., 2015; Sado and Sufa, 2016; Xiao et al., 2017; Abrha et al., 2018; Gereltuya et al., 2018; Saisai et al., 2018; Sun et al., 2018; Guoxu et al., 2019; Neha et al., 2019; Orubu et al., 2019; Demerew and Ahmed, 2020; Xiao et al., 2020; Yi et al., 2020). All studies were conducted in low-income and middle-income countries, including China (6/18), India (3/18), Ethiopia (3/18), Sri Lanka (2/18), Uganda (1/18), Nigeria (1/18), Mongolia (1/18), Guatemala (1/18). Among them, 17 studies (17/18, 94.4%) used the WHO/Health Action International (HAI) medicine price methodology to investigate the accessibility of medicines for children. The WHO/HAI standardized methodology (World Health Organization and Health Action International, 2008) was a research method jointly developed by WHO and HAI in 2000 to standardize studies on the accessibility of medicines. The WHO/HAI standardized methodology was often used for evaluating the accessibility of medicines including medicine availability, price, and affordability. Only one study conducted a cross-sectional survey using a self-formulating questionnaire and its methods to the selecting investigated areas and medicine outlets were not following WHO/HAI standardized methodology. In addition, 15 studies (15/18, 83.3%) selected medicines based on the WHO Model List of Essential Medicines for Children (WHO EMLc). The remaining three studies focused on the WHO recommended priority life-saving medicines for children under five (Nsabagasani et al., 2015; Abrha et al., 2018; Demerew and Ahmed, 2020) (Table 1).

**TABLE 1:** Basic characteristics of included studies.

Study number	Study design	Time	Area	Methods	Single/Multi-center	Number of medicines	Number of medicine outlets	
							Public sector	Private sector
Xiao et al. (2017)	Cross-sectional study	2012/11–2012/12	Shaanxi Province, China	WHO/HAI	Multi-center	28	30	30
Saisai et al. (2018)	Cross-sectional study	2016/11–2016/12	China	WHO/HAI	Multi-center	121	55	0
Guoxu et al. (2019)	Cross-sectional study	2012, 2016	China	WHO/HAI	Multi-center	49	1725	0
Yi et al. (2020)	Cross-sectional study	2017/5/26–2017/6/2	China	WHO/HAI	Multi-center	42	55	0
Xiao et al. (2020)	Cross-sectional study	2019/6–2019/8	Weifang City, Shandong Province, China	WHO/HAI	Multi-center	30	34	37
Sun et al. (2018)	Cross-sectional study	2017/7/10–2017/9/5	Jiangsu Province, China	WHO/HA	Multi-center	40	30	30
Swain et al. (2015)	Cross-sectional study	2010/9/15–2011/4/15	Orissa, India	WHO/HAI	Multi-center	34	79	79
Ravindran et al. (2012)	Cross-sectional study	2011/5–2011/6	East Godabari, Andhra Pradesh, India	WHO/HAI	Multi-center	20	8	7
Neha et al. (2019)	Cross-sectional study	—	New Delhi, India	WHO/HAI	Multi-center	43	7	32
Sado and Sufa (2016)	Cross-sectional study	2015/1	Voliga, Ethiopia	WHO/HAI	Multi-center	22	15	40
Abrha et al. (2018)	Cross-sectional study	2015/12–2016/7	Tigray, Ethiopia	WHO/HAI	Multi-center	27	10	31
Hailu and Mohammed (2020)	Cross-sectional study	2018/11–2019/2	Dessie Town, Ethiopia	WHO/HAI	Multi-center	45	10	0
Balasubramaniam et al. (2011)	Cross-sectional study	2009	Sri Lanka	WHO/HAI	Multi-center	25	40	40
Balasubramaniam et al. (2014)	Cross-sectional study	2009	Sri Lanka	WHO/HAI	Multi-center	25	0	48
Nsabagasani et al. (2015)	Cross-sectional study	2012/6–2012/8	Jinja District,Uganda	—	Multi-center	11	32	0
Orubu et al. (2019)	Cross-sectional study	2016/6–2016/8, 2016/12, 2018/8	Nigeria	WHO/HAI	Multi-center	12	5	12
Gereltuya et al. (2018)	Cross-sectional study	2016/1–2016/8	Mongolia	WHO/HAI	Multi-center	30	11	34
Anson et al. (2012)	Cross-sectional study	2010/4–2010/5	Guatemala	WHO/HAI	Multi-center	27	4	29

## Quality Assessment

The score range of included studies was 5.5–7. Among 18 studies, 17 studies (94.4％, 17/18) were evaluated as high quality, and one study (5.6％, 1/18) was evaluated as medium quality. Four studies (22.2%, 4/18) were considered as unwell-represented samples. The sixth item (Reported the power of test) of the Crombie Scale was not suitable for these included studies, because all these studies were only descriptive statistics (Table 2).

**TABLE 2:** The quality of included studies.

Study number	Design science	Reasonable data collection strategy	Reported sample response rate	Well-represented sample	Reasonable research purposes and methods	Reported the power of test	Reasonable statistical methods	Total score	The level of study	The quality of study
Xiao et al. (2017)	1	1	1	1	1	—	1	7	A	High
Saisai et al. (2018)	1	1	1	1	1	—	1	7	A	High
Guoxu et al. (2019)	1	1	1	1	1	—	1	7	A	High
Yi et al. (2020)	1	1	1	1	1	—	1	7	A	High
Xiao et al. (2020)	1	1	1	1	1	—	1	7	A	High
Sun et al. (2018)	1	1	1	1	1	—	1	7	A	High
Swain et al. (2015)	1	1	1	1	1	—	1	7	A	High
Ravindran et al. (2012)	1	1	1	1	1	—	1	7	A	High
Neha et al. (2019)	1	1	1	1	1	—	1	7	A	High
Sado and Sufa (2016)	1	1	1	1	1	—	1	7	A	High
Abrha et al. (2018)	1	1	1	0	1	—	1	6	A	High
Hailu and Mohammed (2020)	1	1	1	0	1	—	1	6	A	High
Balasubramaniam et al. (2011)	1	1	1	1	1	—	1	7	A	High
Balasubramaniam et al. (2014)	1	1	1	1	1	—	1	7	A	High
Nsabagasani et al. (2015)	0.5	1	1	0	1	—	1	5.5	B	Medium
Orubu et al. (2019)	1	1	1	0	1	—	1	6	A	High
Gereltuya et al. (2018)	1	1	1	1	1	—	1	7	A	High
Anson et al. (2012)	1	1	1	1	1	—	1	7	A	High

## Availability of Medicines for Children

Fourteen studies reported the availability of medicines for children. The availability of medicines was calculated as the percentage (%) of the surveyed outlets where the medicines were found on the day of data collection. In general, as the percentage was less than 30%, it means the availability of medicines was very low; 30–49%, low availability; 50–80%, high availability; >80%, very high availability.

In the public sectors, the availability of OBs ranged from 0 to 52.0% with a median of 24.2%, and the availability of LPGs ranged from 17.0 to 72.6% with a median of 38.1%. In the private sectors, the availability of OBs ranged from 8.9 to 80.0% with a median of 21.2%. The availability of LPGs ranged from 20.6 to 72.2% with a median of 35.9%. In most regions, the availability of OBs in the private sectors was higher than in the public sectors (Table 3).

**TABLE 3:** Availability of medicines for children.

Study number	Area	Public sector			Private sector		
		Medicines availability^a^		Number of medicine outlets	Medicines availability		Number of medicine outlets
		OBs	LPGs		OBs	LPGs	
Xiao et al. (2017)	Shaanxi Province, China	10.8%^b^	27.3%^b^	30	11.9%^b^	20.6%^b^	30
Xiao et al. (2020)	Weifang City, Shandong Province, China	15.8%	44.8%	34	30.3%	36.7%	37
Sun et al. (2018)	Jiangsu Province, China	7.5%	34.2%	30	8.9%	29.4%	30
Yi et al. (2020)	China	33.0%	32.0%	55	—	—	—
Guoxu et al. (2019)	China	1.8%	29.5%	1,725	—	—	—
Sado and Sufa (2016)	Volga, Ethiopia	—	43.0%	15	—	42.8%	40
Abrha et al. (2018)	Tigray, Ethiopia	—	41.9%	10		31.5%	31
Hailu and Mohammed (2020)	Dessie Town, Ethiopia	38.0%^b^	—	10	—	—	—
Swain et al. (2015)	Orissa, India	—	17.0%	79	—	38.5%	79
Ravindran et al. (2012)	East Godavari, Andhra Pradesh, India	32.5%	—	8	37.9%	—	7
Nsabagasani et al. (2015)	Uganda, Jinja District	36.0%	—	32	—	—	—
Anson et al. (2012)	Guatemala	—	46.0%	4	12.0%	35.0%	29
Balasubramaniam et al. (2011)	Sri Lanka	52.0%	—	40	80.0%	—	40
Gereltuya et al. (2018)	Mongolia	—	72.6%	11	—	76.7%	34

a Medicines availability was the percentage of medicine outlets which showed the survey medicine on the survey day.

b This study did not report the overall availability of medicine but report each medicine’s availability respectively, thereby we ranked each medicine’s availability and used the median to represent the overall availability of the medicine.

### Prices of Medicines for Children

Twelve studies reported the price of medicines for children, which were evaluated by the MPR. The MPR was the ratio of the median unit (each tablet, capsule, milliliter, press, etc.) price of a medicine to the International Reference Price (IRP) (Management Sciences for Health, 2015). If MPR <1, it means that the price of the medicine in the survey area was lower than the IRP; if MPR >1, it means that the price of the medicine was higher than the IRP. The IRP of medicines comes from the Guidelines for International Medicine Price Indicator Guide issued by the Management Sciences for Health (MSH) and is updated regularly. If the retail price of medicines in the public sectors does not exceed 1.5 times the IRP, MPR <1.5, or the retail price of medicines in the private sectors does not exceed 2 times IRP, MPR <2, the prices are considered reasonable (Abrha et al., 2018; Sun et al., 2018; Demerew and Ahmed, 2020; Xiao et al., 2020; Yi et al., 2020).

The results showed that the MPR of the OBs in the public sectors and the private sectors was much higher than that of the LPGs. The MPR of the OBs in the public sectors was 5.43 (1.80–25.92) (median (25th percentile of MPR, 75th percentile of MPR)), and in the private sectors was 3.80 (1.00–8.22). The MPR of the LPGs in the public sectors was 1.77 (0.56–7.00), and in the private sectors was 1.54 (0.71–13.10). Meanwhile, the percentages of the OBs and the LPGs in the public sectors with MPR>1.5 were 91.2%, 41.5%, and the percentages of the OBs and the LPGs in the private sectors with MPR>2.0 were 86.5%, 50.3% (Table 4).

**TABLE 4:** Prices of medicines for children.

Study number	Area	Public sector						Private sector					
		OBs			LPGs			OBs			LPGs		
		MPR (P25, P75)^a^	Number of MPR >1.5	Total number of survey medicines	MPR (P25, P75)^a^	Number of MPR >1.5	Total number of survey medicines	MPR (P25, P75)^a^	Number of MPR >2	Total number of survey medicines	MPR (P25, P75)^a^	Number of MPR >2	Total number of survey medicines
Yi et al. (2020)	China	5.43	23	25	1.55	18	36	—	—	—	—	—	—
Xiao et al. (2017)	Shaanxi Province, China	4	3	3	1.1	6	20	3.89	1	2	1.26	5	19
Xiao et al. (2020)	Weifang City, Shandong Province, China	7.76 (3.16, 11.59)	5	6	1.38 (0.6, 5.45)	4	11	6.92 (4.62, 10.34)	8	9	2.68 (1.2, 8.18)	5	10
Sun et al. (2018)	Jiangsu Province, China	2.47–7.70	8	8	1.41–2.12	13	32	5.07–8.22	9	10	1.10–2.24	15	29
Guoxu et al. (2019)	China	11.16	8	8	0.56	12	42	—	—	—	—	—	—
Sado and Sufa (2016)	Volga, Ethiopia	—	—	—	1.18 (0.9, 1.3)	2	12	—	—	—	1.54 (1.23, 2.07)	4	12
Abrha et al. (2018)	Tigray, Ethiopia	—	—	—	1.5 (1.1, 3.6)	4	10	—	—	—	2.7 (1.7, 4.6)	6	10
Hailu and Mohammed (2020)	Dessie Town, Ethiopia	1.80	2	4	—	—	—	—	—	—	—	—	—
Anson et al. (2012)	Guatemala	—	—	—	—	—	—	—	—	—	13.1	15	15
Balasubramaniam et al. (2014)	Sri Lanka	—	—	—	—	—	—	3.70 (0.23, 20)	14	16	1.35 (0.05, 3.75)	5	21
Orubu et al. (2019)	Nigeria	25.92	3	3	7	4	4	—	—	—	—	—	—
Gereltuya et al. (2018)	Mongolia	—	—	—	1.95	20	33	—	—	—	2.45	20	33

a P25, 25th percentile of MPR; P75, 75th percentile of MPR.

### Affordability of Medicines for Children

Eleven studies reported the affordability of medicines for children. The affordability was estimated by using median local prices and the average salary of the lowest-paid unskilled government employee. It was defined as “number of days” wages of a lowest-paid unskilled government worker cost on standard medicine therapy. Following the international standard treatment guidelines, the cost of standard doses medicines to treat a certain disease (a chronic disease in 1 month) was calculated (World Health Organization, 2010). The ratio less than one represented the cost of the medicine less than the minimum wage for 1 day, thus, the medicine was considered affordable. For each medicine, data were collected for both OB. OB was defined as the product that is first authorized worldwide for marketing, and the LPG equivalent was found at each medicine outlet (Cameron et al., 2009).

The results showed that in the public sectors, the percentages of affordable medicines for the OBs and the LPGs were 48.5% (16/33) and 83.6% (97/116), and in the private sectors were 68.8% (11/16) and 72.2% (52/72). The affordability of LPGs in the public sectors and the private sectors was higher than that of OBs. It is notably indicating that the affordability of LPGs was better in most regions, while the costs of OBs were higher, which cost a day wage or more than a day wage (Table 5).

**TABLE 5:** Affordability of medicines for children.

Study number	Area	Public sector				Private sector			
		OBs		LPGs		OBs		LPGs	
		The affordable medicines number^a^	Total number of survey medicines	The affordable medicines number	Total number of survey medicines	The affordable medicines number	Total number of survey medicines	The affordable medicines number	Total number of survey medicines
Xiao et al. (2017)	Shaanxi Province, China	2	3	14	14	2	2	12	12
Yi et al. (2020)	Weifang City, Shandong Province, China	2	4	8	9	4	7	7	7
Sun et al. (2018)	Jiangsu Province, China	6	8	7	8	5	7	6	7
Yi et al. (2020)	China	2	5	4	5	—	—	—	—
Guoxu et al. (2019)	China	2	8	40	42	—	—	—	—
Sado and Sufa (2016)	Volga, Ethiopia	—	—	3	10	—	—	3	10
Abrha et al. (2018)	Tigray, Ethiopia	—	—	7	12	—	—	5	12
Hailu and Mohammed (2020)	Dessie Town, Ethiopia	2	5	—	—	—	—	—	—
Anson et al. (2012)	Guatemala	—	—	—	—	—	—	8	12
Orubu et al. (2019)	Nigeria	—	—	2	4	—	—	—	—
Gereltuya et al. (2018)	Mongolia	—	—	12	12	—	—	11	12

a The affordable medicines number showed the medicine number of days wages to pay for treatment <1 day.

## Discussion

There were 18 studies included in this systematic review. Fifteen studies (15/18, 83.3%) selected medicines based on a core medicines list from the WHO EMLc and a supplementary medicines list from the National Essential Medicines List (NEML). These medicines were selected to represent a series of treatments for commonly acute and chronic conditions that cause substantial morbidity and mortality. In global, regional, and national treatment guidelines, the selected medicines were recommended as first-line options of treatment available for standard formulations and widely used in many countries or regions (The lists of medicines in each survey for children were presented in Supplementary Appendix I).

Seventeen studies (17/18, 94.4%) used the WHO/Health Action International (HAI) medicine price methodology. Before the WHO/HAI standardized methodology was developed, only a few small studies have been investigated in low-income and middle-income countries to assess medicine prices and generate international comparisons. The lack of consistent or reproducible methodologies limits the comparability of these studies’ results and contributes to the vulnerability in criticism (Andersson, 1993). Therefore, a resolution endorsed by the Member States of the WHO developed a standardized method for measuring medicine prices promoting the launch of the WHO/Health Action International (HAI) Project on Medicine Prices and Availability. The WHO/HAI standardized methodology has allowed for the survey of medicine prices and availability in a standardized way, with multiple steps to ensure data quality (such as clearly stipulating how to identify survey areas and survey medicine outlets, select survey medicines, collect and analyze data, etc.). The common list of 30 core medicines with specified dosage forms and strengths allows for more reliable international comparisons and supplementary medicines ensures local relevance at the country level (Cameron et al., 2009). Since the development of WHO/HAI standardized methodology, WHO has worked on a more alternative and sophisticated way for longitudinal data collection with respect to the price and availability of medicines. In 2016, WHO developed an innovative multi-language tool to rapidly collect and analyze data on the price and availability of medicines in health facilities and procurement center–WHO Essential Medicines and Health Products Price and Availability Monitoring Mobile Application (WHO EMP MedMon) This novel tool has improved considering the elements of the WHO/HAI methodology for “Measuring medicine prices, availability, affordability and price components” (WHO, 2018). Moreover, the WHO EMP MedMon App allows users to routinely monitor medicines’ prices and availability in a sustainable, cost-effective, and timely manner. This tool can avoid duplication of efforts and potential manual entry errors by providing photo and barcode capture to identify data when data is collected on paper and then transferred to an electronic format. Furthermore, the tool is customized for any country’s need to collect routine medicines’ data in a fraction of the time and can connect to WHO Medmon’s Power BI platform for interactive analytics and reporting. WHO has tested the application in several pilots, collecting several types of price and availability data, and these results indicate the good feasibility and real-time of this tool (WHO, 2018).

Despite its many strengths, the WHO/HAI methodology also has several limitations. Firstly, differences in quality across products and differences in patent status between countries are not accounted for Ridley (2005). Such as, in India, there are a large number of generic manufacturers and the pricing regulations in force do not recognize medicine patents. Secondly, the reliability of the median price ratio (MPR) as a metric for comparison depends on each medicine’s median international reference price (IRP) which is susceptible to the number of supplier prices. When few supplier prices are available or when the buyer price is used as a substitute, MPR results can be skewed by a particularly high or low IRP. Concurrently, the survey method is more complicated and requires much manpower, material, and time. In addition, most of the medicine availability data obtained are the availability of medicines in the sectors on the survey day, and it cannot reflect each of these sectors’ monthly or annual average supply of medicines. The results in our review indicated that the availability of most medicines for children was low in the public sectors and private sectors, and the prices of medicines exceeded IRP. The public sector’s availability of medicines for children was consistently low, which could be due to high demand but insufficient supply, as general public sectors’ medicines are available for free or at low cost. This could be due to a combination of factors further, such as inadequate funding, lack of incentives for maintaining stocks, inability to forecast accurately, inefficient distribution systems, or leakage of medicines for private resale (Cameron et al., 2009). The availability in the private sector was slightly higher, but in many countries, it was still low, and the higher private sector prices could hinder access to medicines in children. Although the policy on the accessibility of medicines for children has improved in recent years, there may be a lack of compulsory regulations on medicine procurement and pricing. The consistency, system, and accuracy of the policy need to be improved.

### Limitations

Firstly, this review only included Chinese and English articles, which could have a language bias. Also, some of the studies included in this review were conducted 5 years ago. Many countries have taken measures on improving the accessibility of medicines for children (such as the medical insurance catalogue and the national essential medicine catalogue will be revised regularly). Therefore, the previous survey results could not be fully consistent with the current accessibility of medicines in these regions. Importantly, the medicine availability data is the availability of medicines in surveyed sectors on the survey day, which could not truly reflect the monthly or annual average medicine supply of these sectors. It also needs to considered that most of the included studies adapted the WHO/HAI standardized methodology to investigate and evaluate the medicine price and affordability, in which only a small number of medicines could obtain the data of price and affordability, so these data may not accurately reflect the real situation of local children’s medicine prices and affordability. Lastly, all of the included studies investigated the accessibility of medicines for children in low-income and middle-income countries, and these survey results could not apply to high-income countries.

### Recommendations

Based on the results of this review, the data confirmed that substantial opportunities exist to increase availability, lower prices, and improve the affordability of children’s medicines in all regions and countries. Countries should establish a regular investigation and report system for the accessibility of children’s medicines to regularly investigate the availability, prices, and affordability of medicines. Routinely monitored and timely report the investigation and evaluation results to relevant departments is crucial. Medicine prices, availability, and affordability should be surveyed and reported at least every 2 years by using the WHO/HAI methodology (Cameron et al., 2009). These data provided for countries can develop, implement, assess, and adjust national policies and medicine prices to improve the availability and affordability of children’s medicines. The WHO MEDMON tool may be an appropriate tool to help any country to create its own routine data collection systems for real-time monitoring, and to assist the government to assess policy interventions’ effect. In the third WHO Fair Pricing Forum on reaching fairer prices of medicines and health tools ended on 22 April 2021, WHO announced that later this year, they would launch its updated MEDMON to monitor availability and prices of health products in several countries. Simultaneously, WHO would continue to support these countries with the development of national or regional price monitoring systems and hosting further webinar and training to strengthen policy-makers’ capacity (WHO, 2021). Further, encouraging the use of those low-cost generic medicines with the dominant efficacy may be able to significantly reduce medical expenditures and increase medicine affordability.

## Conclusion

The availability of medicines for children in both public sectors and private sectors in low-income and middle-income countries is low. The MPR of OBs is higher than that of LPGs, and the LPGs is implicated with the better affordability.

## Data Availability

The raw data supporting the conclusion of this article will be made available by the authors, without undue reservation.
