# Supplementary material for: Accessibility of Medicines for Children: A Systematic Review
Source: Front Pharmacol. 2021 Aug 5;12:691606. doi: 10.3389/fphar.2021.691606 (PMC8375435; doi:10.3389/fphar.2021.691606)
Supplement: Supplementary file 1 [file DataSheet1.PDF]

Appendix I

Lists of access to medicines survey for children in each included study

Study ID	Survey medicines name	Strength	Dosage form
Yi Dai, 2020	Cefazolin	1.0g	injection
	Ceftriaxone	0.5g	injection
	Cefuroxime	0.75g	injection
	Cefuroxime	0.25g	tablet
	Cefotaxime	0.5g	injection
	Ceftazidine	1.0g	injection
	Vancomycin	0.5g	injection
	Fluconazole	2.0mg/ml	injection
	Fluconazole	50mg	tablet/capsule
	Acyclopedia	0.25g	injection
	Acyclovir	0.2g	tablet
	Nystatin	500 000U	tablet
	Metronidazole	0.5 g/100ml	injection
	Metronidazole	0.2g	tablet
	Azithromycin	0.25g	tablet
	Isoniazid	100 mg	tablet
	Rifampin	150 mg	tablet
	Carbamazepine	0.2g	tablet
	Phenobarbital	30mg	tablet
	Sodium Valproate	0.5g	tablet
	Enalapril	5mg	tablet
	Digoxin	50μg/ml	solution
	Digoxin	0.25 mg	tablet
	Salbutamol	100μg/dose	Inhaler
	Monroust	5 mg	tablet
	Cyclosporine	25 mg	tablet/capsule
	Cytarabine	100 mg	injection
	Cytarabine	500mg	injection
	Ifosfamide	1g	injection
	Methotrexate	2.5 mg	tablet
	Vincristine	1mg	injection
	Omeprazole	10 mg	tablet
	Omeprazole	20 mg	tablet
	Ranitidine	150mg	tablet/capsule
	aspirin	0.1g	tablet
	Mupirocin	0.02	ointment
	2% miconazole ointment	0.02	ointment
	Loratadine	10 mg	tablet
	Loratadine	1mg/ml	solution
	Prednisolone	5mg	tablet

	Ibuprofen	200 mg/5ml	solution
	Levothyroxine	50μg	tablet
	Amitriptyline	/	/
	Amoxicillin	/	/
	aspirin	/	/
	Azithromycin	/	/
	Amoxicillin and Clavulanate Potassium	/	/
	Omeprazole	/	/
	Amlodipine	/	/
	Phenytoin sodium	/	/
	Beclomethasone dipropionate	/	/
	Furosemide	/	/
	Metformin	/	/
	Erythromycin	/	/
	Glibenclamide	/	/
	Captopril	/	/
	Ranitidine	/	/
	Salbutamol	/	/
	Simvastatin	/	/
	Enalapril	/	/
	Clarithromycin	/	/
	Isoniazid	/	/
Guoxu Wei, 2019	Hydrocortisone	/	/
	Nifedipine	/	/
	Diphenhydramine	/	/
	Digoxin	/	/
	Isosorbide dinitrate	/	/
	Ambroxol	/	/
	Montmorillonite	/	/
	Vitamin K1	/	/
	Compound Amino Acid	/	/
	Acetaminophen	/	/
	Carbamazepine	/	/
	Ceftriaxone	/	/
	Sodium Valproate	/	/
	Levofloxacin	/	/
	Promethazine	/	/
	Aminophylline	/	/
	Allopurinol	/	/
	Ibuprofen	/	/
	Atenolol	/	/
	Metronidazole	/	/
	Ciprofloxacin	/	/
	Cefuroxime	/	/

	Cephalexin	/	/
	Albendazole	/	/
	Diclofenac Sodium	/	/
	Loratadine	/	/
	Chlorpheniramine	/	/
	Sulfadiazine	/	/
	Calcium gluconate	/	/
	Salbutamol	/	/
	Ceftriaxone	/	/
	Ibuprofen	/	/
	Metformin	/	/
	Amoxicillin	/	/
	Sodium Valproate	/	/
Saisai Li, 2018	Albendazole	/	/
	Metronidazole	/	/
	Enalapril	/	/
	Hydrochlorothiazide	/	/
	Cephalexin	/	/
	Diazepam	/	/
	Ranitidine	/	/
	Amitriptyline	/	/
	Adrenaline	1 mg/ml	injection
	Amoxicillin	250 mg	tablet/capsule
	Azithromycin	250 mg	tablet/capsule
	Calamine	100 ml	solution
	Carbamazepine	200 mg	tablet
	Cefazolin	1 g	injection
	Ceftriaxone	1 g	injection
	Diazepam	10 mg/2 ml	injection
	Fluconazole	0.2 g/100 ml	injection
	Furosemide	20 mg/2 ml	injection
	Hydrochlorothiazide	25 mg	tablet
Xiao Wang, 2020	Ibuprofen	200 mg	tablet/capsule
	Isoniazid	100 mg	tablet
	Levothyroxine	50 μg	tablet
	Loratadine	10 mg	tablet/capsule
	Mannitol	50 g/250 ml	injection
	Metformin	500 mg	tablet/capsule
	Metronidazole	200 mg	tablet/capsule
	Midazolam	5 mg/ml	injection
	Acetaminophen	500 mg	tablet
	Phenobarbital	30 mg	tablet
	Phenytoin sodium	50 mg	tablet
	Piperacillin and tazobactam	4.5 g	injection

	Salbutamol	100 mcg/dose	Inhaler
	Albendazole	200 mg	tablet
	aspirin	100 mg	tablet
	Cefuroxime	750 mg	injection
	Digoxin	0.5 mg/2 ml	injection
	Prednisone	5 mg	tablet
	Vitamin K1	10 mg/ml	injection
Edao Sado, 2016	Amoxicillin	125 mg/5 ml	Suspension
	Amoxicillin	250 mg/5 ml	Suspension
	Amoxicillin + Clavulanic Acid	125 mg + 31.25 mg/5 ml	Suspension
	Amoxicillin + Clavulanic Acid	250 mg + 62.5 mg/5 ml	Suspension
	Artesunatea	60 mg	Vial
	Artemether + Lumefantrinea	20 mg + 120 mg	Dispersible tab
	Chloramphenicol	1 gm	Vial
	Carbamazepine	100 mg/5 ml	Syrup
	Ceftriaxone	500 mg	Vial
	Cotrimoxazole	40 mg + 200 mg/5 ml	Suspension
	Diazepam	5 mg/ml	Ampoule
	Gentamicin	20 mg/2 ml	Ampoule
	Ibuprofen	100 mg/5 ml	Suspension
	ORS	To make 500 ml	Powder
	Paracetamol	120 mg/5 ml	Syrup
	Paracetamol	125 mg	Suppository
	Penicillin G	1 million IU	Vial
	Procaine Penicillin G	4 million IU	Vial
	Salbutamol	100 mcg/dose	Inhaler
	Vitamin A	50000 U	Capsule
	Zinc Phosphatea	20 mg	Dispersible tab
Solomon Abrha, 2018	Procaine Penicillin G	4 million IU	Vial
	Amoxicillin	250 mg	capsule
	Amoxicillin	500 mg	capsule
	Gentamicin	40 mg/mL	injection
	Ceftriaxone	250 mg	powder for injection
	Ceftriaxone	1 g	powder for injection
	Morphine	100 mg	granules capsule
	Morphine	60 mg	granules capsule
	Morphine	30 mg	granules capsule
	Morphine	20 mg	granules capsule
	Morphine	200 mg	granules capsule
	Morphine	10 mg/mL	injection
	Morphine	10 mg/5 mL	oral liquid
	Vitamin A	100000 IU	capsule
	Vitamin A	200000 IU	injection
	Paracetamol	100 mg	tablet

	Paracetamol	120 mg/5 mL	oral liquid
	Paracetamol	200 mg	suppository
	Oxygen medicinal gas	/	/
	Procaine benzyl penicillin	1 g	powder for injection
	Artemisinin combination therapy tablet	/	tablet
	Artesunate	50–200 mg	injection
	Artesunate	50–200 mg	suppository
	Zinc	20 mg	tablet
	ORS, in sachets	/	powder
	Ampicillin	250 mg	powder for injection
	Ampicillin	500 mg	powder for injection
	Ampicillin	1 g	powder for injection
	Amoxicillin	250 mg	capsule/tablet
	Amoxicillin/Clavulanicacid	125 mg+31.25 mg/5 ml	suspension
	Azithromycin	250 mg	tablet
	Benzylpenicillin	1 million IU	injection
	Calamine	100 ml	lotion
	Carbamazepine	200 mg	tablet
	Cefazolin	1 g	injection
	Ceftriaxone	250 mg	injection
	Chloramphenicol	250 mg	tablet
	Chlorpheniramine	4 mg	tablet
	Diazepam	5 mg/ml	injection
	Fluconazole	50 mg	capsule
	Ibuprofen	200 mg	tablet
Xiao Wang, 2017	Isoniazid	100 mg	tablet
	Morphine	10 mg	tablet
	Oralrehydrationsolution	500 ml	oralsolution
	Paracetamol	500 mg	tablet
	Phenobarbital	30 mg	tablet
	Phenytoin	50 mg	tablet
	Procainepenicillin	600 mg	injection
	Salbutamol	100 mcg/dose	inhaler
	Albendazole	200 mg	tablet
	Aminophylline	25 mg/ml	injection
	Amoxicillin/Clavulanicacid	250 mg+125 mg	tablet
	Beclomethasone	50 mcg/ dose	inhaler
	Phenobarbital	100 mg/ml	injection
	VitaminA	25 000 IU	capsule
	VitaminB6	50 mg/ml	injection
	Asparaginase	10 000 IU	powder inj
Neha Faruqui, 2019	Bleomycin	15 mg	powder inj
	Carboplatin	150 mg/15 mL	inj
	Carboplatin	450 mg/45 mL	inj

	Carboplatin	50 mg/5 mL	inj
	Carboplatin	600 mg/60 mL	inj
	Carboplatin	no specific strength	/
	Cisplatin	100 mg/100 mL	inj
	Cisplatin	50 mg/50 mL	inj
	Cisplatin	no specific strength	/
	Cyclophosphamide	500 mg in vial	powder inj
	Cyclophosphamide	25 mg	tab
	Cytarabine	100 mg in vial	powder inj
	Dacarbazine	100 mg in vial	powder inj
	Dactinomycin	500 µg in vial	powder inj
	Daunorubicin	50 mg in vial	powder inj
	Dexamethasone	2 mg/5 mL	oral liquid
	Doxorubicin	10 mg	powder inj
	Doxorubicin	50 mg	powder inj
	Doxorubicin	no specific strength	/
	Etoposide	100 mg	cap
	Etoposide	100 mg	inj
	Ifosfamide	500 mg	powder inj
	Ifosfamide	2 g vial	powder inj
	Ifosfamide	1 g	powder inj
	Ifosfamide	no specific strength	Powder inj
	Mercaptopurine	50 mg	tab
	Methotrexate	50 mg	powder inj
	Methotrexate	2.5 mg	tab
	Paclitaxel	6 mg/mL	powder inj
	Prednisolone	5 mg/mL	oral liquid
	Prednisolone	25 mg	tab
	Prednisolone	5 mg	tab
	Prednisolone	no specific strength	tab
	Thioguanine	40 mg	solid oral dosage form
	Vinblastine	10 mg (sulfate) in vial	powder inj
	Vincristine	1 mg	powder inj
	Vincristine	5 mg	powder inj
	Vincristine	no specific strength	powder inj
	Amoxicillin	250 mg	cap/tab
	Ciprofloxacin	500 mg	cap/tab
	Metformin	500 mg	cap/tab
	Omeprazole	20 mg	cap/tab
	Salbutamo	100 mcg/dose	Inhaler
	Beclomethasone	100 mcg/dose	Inhaler
Anson A, 2012	Zinc	20 mg	Dispersible tab
	Oral Rehydration Solution	500 ml	Solution
	Oral Rehydration Solution	To make 500ml	powder

	Amoxicillin	100 mg/ml	Pediatric drops
	Amoxicillin	125 mg/5ml	Suspension
	Amoxicillin	250 mg/5ml	Suspension
	Amoxicillin + Clavulanic Acid	125 mg + 31.25mg/5ml	Suspension
	Amoxicillin + Clavulanic Acid	250 mg + 62.5mg/5ml	Suspension
	Benzathine Penicillin G	1.2 M units/vial	Injection
	Ceftriaxone	500 mg/vial	Injection
	Chloramphenicol	1 g/vial	Injection
	Cotrimoxazole (Trimethoprin + Sulfamethoxazole)	8 + 40 mg/ml	Suspension
	Gentamicin	10mg/ml	Injection
	Procaine Penicillin G	4 M units/vial	Injection
	Primaquine	15mg	Cap/tab
	Ibuprofen	200 mg	Cap/tab
	Paracetamol	25 mg/ml	syrup/susp
	Morphine Sulfate	10mg/ml	Injection
	Vitamin A	50 000 units	Cap/tab
	Isoniazid	100 mg	Cap/tab
	Ferrous Salt	125 mg/5ml	Suspension
	Diazepam	5 mg/ml	Injection
	Carbamazepine	100 mg/5ml	Suspension
	Phenobarbital	3mg/ml	Oral liquid
	Phenytoin	25 or 30mg/5ml	Suspension
	Spacer	n/a	n/a
Hailu Abel, 2020	Amoxicillin	250 mg	Dispersible, scored tablets
	Amoxicillin	500 mg	Dispersible, scored tablets
	Ampicillin	500 mg	powder for injection
	Ceftriaxone	1 g	powder for injection
	Ceftriaxone	500 mg	powder for injection
	Ceftriaxone	250 mg	powder for injection
	Gentamicin	40 mg/ml(20 mg/ml)	injection
	Oxygen	/	medicinal gas
	ORS	sachets of 500 ml and 1 l	powder
	ORS	sachets of 1 l	powder
	Zinc	20 mg	scored dispersible tablet
	Artesunate	50–200 mg	injection
	Lamivudine + nevirapine + zidovudine	30 mg + 50 mg + 60 mg	tablet
	Vitamin A	100000 IU	capsule
	Vitamin A	200000 IU	capsule
	Morphine	10 mg/mL	granules injection
	Oxytocin	10 IU in 1ml ampoule	injection
	Sodium chloride	solution	injectable solution 0.9% isotonic
	Sodium lactate compound((Ringer’s	/	injectable

	lactate))		
	Magnesium sulfate	500 mg/ml	Injection(Ampoule)
	Calcium gluconate	100 mg/ml	Injection(Ampoule)
	Hydralazine	20 mg	powder for injection
	Methyldopa	250 mg	tablet
	Metronidazole	500 mg	injection
	Mifepristone + misoprostol	200 mg + 200 mg	tablet
	Azithromycin	250 mg	capsule
	Azithromycin	500 mg	capsule
	Nifedipine	10 mg	immediate release capsule
	Dexamethasone	4 mg	injection
	Tetanus vaccine	/	/
	Oral contraceptives (pack of 2)	/	/
	Intrauterine devices and barrier methods of contraception(e.g. condoms)	/	/
	Implantable contraceptives estradiol cypionate+ medroxyprogesterone acetate	/	/
	Efavirenz + lamivudine + tenofovir	600 mg + 300mg + 300 mg	/
	Artemether–Lummefantrine	/	/
	Artesunate	50–200 mg	injection
	Chloroquine	250 mg	/
	Oral polio vaccine	/	/
	Bacille Calmette-Guérin	/	/
	Pneumococcal conjugate vaccine	/	/
	Measles vaccine	/	/
	Tetanus Toxoid Vaccine	/	/
	Penta Valent	/	/
	Intractable Polio vaccine	/	/
	Rotavirus vaccine	/	/
	Albendazole	200 mg	tablet
	Amoxicillin	250 mg	capsule
	suspension	100 ml	125 mg
	injection	1 vial	25 mg/ml
	Amoxicillin_Clavulanic acid	125 mg + 125 mg	tablet
	Amoxicillin_Clavulanic acid	125 mg + 31.25 mg	suspension
Gereltuya Dorj, 2018	Amoxicillin_Clavulanic acid	250 mg + 62.50 mg	suspension
	Azithromycin	250 mg	capsule
	Azithromycin	200 mg/5 ml	suspension
	Beclomethason	50 mg/day	inhaler
	Benzylpenicillin	600 mg = 1 million IU	injection
	Carbamazepine	100 mg	tablet
	Carbamazepine	100 mg/5 ml	suspension
	Cefazolin	1 g,vial	injection
	Ceftriaxone	500 mg, vial	injection

	Chloramphenicol	250 mg	tablet
	Chloramphenicol	1 g,vial	injection
	Chlorpheniramine	4 mg	tablet
	Clarithromycin	125 mg/ 5 ml	suspension
	Clarithromycin	125 mg	tablet
	Cotrimoxazole	100 mg + 20 mg (also expressedas 400 mg + 80 mg)	tablet
	Cotrimoxazole	100 ml	suspension
	Diazepam	5 mg	tablet
	Ferrous salt	30 mg/5 ml	suspension
	Fluconazole	150 mg	capsule
	Gentamycin	10 mg/ml	injection
	Ibuprofen	2 mg	tablet
	Ibuprofen	100 ml	suspension
	Isoniazide	100 mg	tablet
	Morphine	10 mg	tablet
	Morphine	10 mg/5 ml	oral solution
	ORS	500 ml	sachet
	Paracetamol	250 mg	tablet
	Paracetamol	125 mg	suppository
	Paracetamol	250 mg	suppository
	Paracetamol	125 mg	suspension
	Phenobarbital	30 mg	tablet
	Phenobarbital	100 mg/ml	injection
	Phenytoin	50 mg	tablet
	Phenytoin	25 mg,30 mg/5 ml	suspension
	Procain penicillin	1 g, vial	injection
	Salbutamol	100mcg	inhaler
	Vitamin A	100.000 IU	capsule
	Vitamin B6	50 mg/ml	injection
	Zinc	20 mg	tablet
	Device	/	/
Xavier Nsabagasani, 2015	Amoxicillin	250 mg	Dispersible, scored tablets
	Amoxicillin	500 mg	Dispersible, scored tablets
	Amoxicillin	500 mg	Powder for injection
	Amoxicillin	1 g	Powder for injection
	Ceftriaxone	500 mg	Powder for injection
	Ceftriaxone	1 g	Powder for injection
	Gentamycin	40 mg/ml	Injection
	Gentamycin	20 mg/ml	Injection
	Oral Rehydration Salt (ORS)	Sachets of 200 ml	Powder
	Oral Rehydration Salt (ORS)	Sachets of 500 ml	Powder
	Oral Rehydration Salt (ORS)	Sachets of 1000 ml	Powder
	Zinc sulphate	20 mg	Dispersible tablet

	Artemesinin combination therapy (ACT)	/	Dispersible tablets
	Artesunate	50–200 mg	Injection
	Artesunate	50–200 mg	Rectal
	Procaine benzyl penicillin	1 g	injection
	Amoxicillin	125 mg / 5 ml (100 ml)	Suspension
	Amoxicillin	250 mg	Capsule/Tablet
	Amoxicillin + clavulanic acid	125 mg + 31.25 mg / 5 ml (100 ml)	Suspension
	Beclometasone – MDI	50 microgram/dose (200 doses)	Inhaler
	Carbamazepine	100 mg / 5 ml (100 ml)	Suspension
	Ceftriaxone	1 gram (vial)	Injection
	Chlorphenamine	2 mg / 5 ml (100 ml)	Syrup
	Clotrimazole	1% (15 g tube)	Topical cream
	Cloxacillin	125 mg / 5 ml (100 ml)	Syrup
	Co-trimoxazole	200 mg + 40 mg / 5 ml (100 ml)	Suspension
	Diazepam	5 mg / ml (2 ml ampoule)	Injection
Balasubramaniam R, 2011	Diethylcarbamazine citrate	50 mg	Tablet
	Domperidone	5 mg / 5ml (100 ml)	Syrup
	Erythromycin	125 mg / 5 ml (100 ml)	Syrup
	Ferrous salt	30 mg / ml (250 ml)	Suspension
	Ibuprofen	100 mg / 5ml (100 ml)	Syrup
	Mebendazole	100 mg (6 tablets)	Chewable tablet
	Mebendazole	100 mg / 5 ml (30 ml)	Syrup
	Metronidazole	200 mg	Tablet
	Oral rehydration salt	Packet to make 1 litre of solution	Powder
	Paracetamol	120 mg / 5 ml (100 ml)	Syrup
	Paracetamol	500 mg	Tablet
	Salbutamol – MDI	100 microgram / dose (200 doses)	Inhaler
	Salbutamol	0.5% (10 ml)	Respiratory solution
	Vitamin C	100 mg	Tablet
	Amoxicillin	125 mg / 5 ml (100 ml)	Suspension
	Amoxicillin	250 mg	Capsule/Tablet
	Amoxicillin + clavulanic acid	125 mg + 31.25 mg / 5 ml (100 ml)	Suspension
Balasubramaniam R, 2014	Beclometasone – MDI	50 microgram/dose (200 doses)	Inhaler
	Carbamazepine	100 mg / 5 ml (100 ml)	Suspension
	Ceftriaxone	1 gram (vial)	Injection
	Chlorphenamine	2 mg / 5 ml (100 ml)	Syrup
	Clotrimazole	1% (15 g tube)	Topical cream

	Cloxacillin	125 mg / 5 ml (100 ml)	Syrup
	Co-trimoxazole	200 mg + 40 mg / 5 ml (100 ml)	Suspension
	Diazepam	5 mg / ml (2 ml ampoule)	Injection
	Diethylcarbamazine citrate	50 mg	Tablet
	Domperidone	5 mg / 5ml (100 ml)	Syrup
	Erythromycin	125 mg / 5 ml (100 ml)	Syrup
	Ferrous salt	30 mg / ml (250 ml)	Suspension
	Ibuprofen	100 mg / 5ml (100 ml)	Syrup
	Mebendazole	100 mg (6 tablets)	Chewable tablet
	Mebendazole	100 mg / 5 ml (30 ml)	Syrup
	Metronidazole	200 mg	Tablet
	Oral rehydration salt	Packet to make 1 litre of solution	Powder
	Paracetamol	120 mg / 5 ml (100 ml)	Syrup
	Paracetamol	500 mg	Tablet
	Salbutamol – MDI	100 microgram / dose (200 doses)	Inhaler
	Salbutamol	0.5% (10 ml)	Respiratory solution
	Vitamin C	100 mg	Tablet
Ebiowei SF Orubu, 2019	digoxin	/	/
	dopamine	/	/
	enalapril	/	/
	furosemide	/	/
	hydrochlorothiazide	/	/
	mannitol	/	/
	spironolactone	/	/
Ravindran L, 2012	Amoxicillin	/	Suspension
	Amoxicillin + clavulanic acid	/	Suspension
	Ceftriaxone	/	Injection
	Cotrimoxazole suspension Ferous salt	/	Suspension
	ORS sachet	/	Powder
	Paracetamol	/	syrup
	Salbutamol	/	inhaler
Xiaoluan Sun, 2018	Aciclovir	200mg	Cap/tab
	Amoxicillin	250mg	Cap/tab
	Amoxicillin/clavulanic acid	125/31.25mg/5ml	Suspen
	Azithromycin	250mg	Cap/tab
	Calamine	100mL	Lotion
	Calcium gluconate	100mg/mL	Ampoule
	Carbamazepine	200mg	Cap/tab
	Ceftriaxone	1g	Phial
	Ceftazidime	1g	Phial
	Clarithromycin (sustained-release)	500mg	Cap/tab

	Clindamycin	150mg	Cap/tab
	Diazepam	5mg/mL	Ampoule
	Fluconazole	50mg	Cap/tab
	Folic Acid	5mg	Cap/tab
	Furosemide	10mg/mL	Ampoule
	Hydrochlorothiazide	25mg	Cap/tab
	Hydrocortisone	100mg	Phial
	Ibuprofen	200mg	Cap/tab
	Loratadine	10mg	Cap/tab
	Miconazole nitrate	0.02	Cream
	Mupirocin	0.02	Cream
	Omeprazole (enteric-coated)	20mg	Cap/tab
	Paracetamol	500mg	Cap/tab
	Phenobarbital	30mg	Cap/tab
	Phenytoin	100mg	Cap/tab
	Propylthiouracil	50mg	Cap/tab
	Ranitidine	150mg	Cap/tab
	Salbutamol	100mcg/dose	Inhaler
	Sodium valproate	200mg	Cap/tab
	Aminophylline	100mg	Cap/tab
	Amoxicillin/clavulanic acid	1000/200mg	Phial
	Cefuroxime	250mg	Cap/tab
	Chlorphenamine maleate	4mg	Cap/tab
	Dexamethasone	5mg/mL	Ampoule
	Clarithromycin	250mg	Cap/tab
	Ibuprofen	100mg/5mL	Suspen
	Phenobarbital	100mg/mL	Ampoule
	Vitamin B6	50mg/mL	Ampoule
	Vitamin C	100mg	Cap/tab
	Sodium valproate (sustained-release)	500mg	Cap/tab
Swain T, 2015	Albendazole	200 mg/5 ml	suspension
	Amoxicillin	125 mg/ml	suspension
	Amoxicillin	250 mg	dispersible scored tablet
	Amoxicillin+clavulanic acid	125mg+31.25mg	dry syrup
	Amoxicillin+clavulanic acid	250 mg +125 mg	dispersible kid forte,FC tablet
	Artemether+lumefantrine	20mg+120mg	dispersible tablet
	Beclomethasone	100 µg/dose	inhaler
	Benzyl benzoate	25%	lotion
	Benzylpenicillin	600 mg =1 milon IU	injection
	Carbamazepine	100 mg/5 ml	suspension
	Carbamazepine	100 mg	chewable tablet
	Chloramphenicol	500 mg/vial	iniecton
	Chloroquine	50 mg/5 ml	suspension

Co-trimoxazole	100 mg + 20mg (aso expressed as 400mg＋80 mg)	dispersible tablet
Diazepam	5 mg/ml	rectal solution
Ferrous sulfate	50mg Fe/5ml	suspension
Gentamycin	10 mg/ml	injection
lbuprofen	200 mg	tablet
lsoniazid + rifampicin + pyrazinamide	50 mg+100 mg+300mg	dispersibletablet
ORS	200 ml	sachet
ORS	1L	achet
Paracetamol	120mg/5ml or 125 mg/5ml	suspension
Paracetamol	250 mg	scored tablet
Phenobarbital	200 mg/ml	injection
Phenytoin	25 or 30 mg/ml	suspension
Procaine penicillin	1g=1milion IU	injection
Salbutamol	100 mcg/dose	inhaler
Vitamin A	25 000 IU	capsule
Zinc.	20 mg	dispersible tablet
Prednisolone	5 mg/5 ml	suspension
Azithromycin	250 mg	dispersible tablet
Ofloxacin	200 mg	tablet
Ondansetron	2 mg/5 ml	syrup/suspension
Valproic acid	200 mg/5 ml	oral liquid
